# An Exploratory Clinical Study on an Automated, Speed-Sensing Treadmill Prototype With Partial Body Weight Support for Hemiparetic Gait Rehabilitation in Subacute and Chronic Stroke Patients

**DOI:** 10.3389/fneur.2020.00747

**Published:** 2020-07-24

**Authors:** Karen Chua, Wei Sheong Lim, Pang Hung Lim, Chien Joo Lim, Chuan Mien Hoo, Kuang Chua Chua, Johnny Chee, Wai Sing Ong, Weidong Liu, Chin Jung Wong

**Affiliations:** ^1^Department of Rehabilitation Medicine, Centre of Advanced Rehabilitation Therapeutics, Tan Tock Seng Hospital Rehabilitation Centre, Singapore, Singapore; ^2^Clinical Research & Innovation Office, Tan Tock Seng Hospital, Singapore, Singapore; ^3^School of Engineering, Ngee Ann Polytechnic, Singapore, Singapore

**Keywords:** stroke, hemiplegia, rehabilitation, speed-sensing, body weight supported treadmill training

## Abstract

Impairments in walking speed, capacity, and endurance are commonly seen after stroke. Treadmill training improves endurance and gait speed. However, the lack of variable training speed and automated speed progression increases the risk of backward displacement and falling. An automated, speed-sensing treadmill prototype with partial body weight support, the Variable Automated Speed and Sensing Treadmill II (VASST II), was tested in an outpatient rehabilitation setting. Eleven subacute or chronic hemiplegics who could ambulate at > 0.2 m/s for >50 m participated in the study. All subjects underwent physiotherapist-supervised training on VASST II for 60 min daily, 3 times per week, for 5 weeks (total 15 h). Outcome measures at Week 3 (mid-VASST II training), Week 6 (post-VASST II training), Week 12 (first follow-up), and Week 24 (second follow-up) included the 6 minute walk test (6 MWT), 10 meter walk test (10 MWT), Berg Balance Scale (BBS) score, and Functional Ambulation category (FAC) score. User acceptability of VASST II for both study subjects and physiotherapists were also assessed. All subjects [median (IQR) age: 53.0 (22) years; median (IQR) duration post-stroke: 524 (811) days] completed VASST II training. At baseline, mean ± SD 6 MWT was 114 ± 50.9 m; mean ± SD 10 MWT was 0.37 ± 0.18 m/s; mean ± SD BBS score was 40 ± 10; and, mean ± SD FAC score was 4 ± 1. At Week 6, there were significant improvements in the 6 MWT [158.91 ± 88.69 m; *P* = 0.003], 10 MWT [0.49 ± 0.30 m/s; *P* = 0.016], and BBS score [42 ± 10; *P* = 0.003]. Improvements in 6 MWT and BBS scores were sustained at Week 24, but not in the 10 MWT. No VASST II-training related falls were reported. All subjects rated their VASST II training positively and indicated that it improved their current walking ability. VASST II training was effective, feasible, and safe in patients with subacute or chronic post-stroke hemiparetic gait, with sustained gains in distance walked (6 MWT) and functional balance (BBS score) up to 19 weeks post-intervention.

## Introduction

Stroke remains a leading cause of death and disability globally. Findings from the Global Burden of Disease Study 2017 indicate that stroke was the third leading cause of mortality (accounting for over 6.1 million deaths), and one of 5 leading causes of morbidity, accounting for 132 million disability- adjusted life-years (DALYs) worldwide in 2017 (1, 2). Hemiparetic weakness affects over 70% of stroke survivors (3). Despite best efforts at rehabilitation, walking dysfunction persists in up to 50% of patients 6 months post-stroke, resulting in <20% of stroke survivors going on to achieve community ambulation (4, 5). Such reductions in walking capacity contribute to the longer-term secondary complications of stroke such as functional decline, cardiovascular disease, deconditioning, and osteoporosis (6–8).

Minimizing impairments and maximizing function are key goals of stroke rehabilitation programmes (9). Data from randomized trials support the effectiveness of task-specific walking interventions in improving walking distance, gait speed, and overall balance in the first year post- stroke (10, 11). To date, several meta-analyses of controlled randomized trials indicate that a variety of motor rehabilitation strategies such as electromechanical gait trainers, partial body weight supported treadmill training (BWSTT) and speed-dependent treadmill training (STT) are beneficial for achieving gains in walking speed and distance, when compared to overground walking training (12–15). Manual treadmill training supervised by physiotherapists has been shown to be a useful technique for improving the gait speed and walking distance for stroke patients (16–18). Treadmill training is postulated to confer beneficial locomotor benefits by enhancing repetitive stepping practice and task-specific training, compared to traditional overground training sessions supervised by physiotherapists (5). This is further corroborated by the findings of a systematic review of studies involving ambulatory sub-acute/chronic stroke patients [Functional Ambulation Category (FAC) score ≥3 and baseline walking speed ≥0.2 m/s], whereby treadmill training improved gait speed by 0.14 m/s [measured by the 10 meter walk test (10 MWT)] and distance walked by 40 m [measured by the 6 minute walk test (6 MWT)] immediately post-intervention (12, 19). Both these gains met their respective minimal clinically important differences (MCIDs)—the MCID range for substantial meaningful change in gait speed is 0.14–0.16 m/s and the MCID range for meaningful improvement in distance walked is 34.4–44 m (20–23). Additionally, these improvements were sustained beyond the intervention period.

Speed-dependent treadmill training (STT) has also been explored as an augmented approach to standard treadmill training based on the principle of sports training i.e., training at speeds below a person's maximum possible speed, does not lead to significant improvements in gait speed (18). Partial body weight supported treadmill training (BWSTT) is another gait re-training strategy using treadmills which involves the use of a harness system to support a percentage of a patient's body weight in order to reduce the load-bearing on the lower extremities and provide added stability (24). In general, BWSTT has been shown to improve gait speed, balance and functional status of stroke patients in both clinic and community settings. Several studies have demonstrated that BWSTT, with up to 40% BWS at the beginning of training, has a far greater effect on gait parameters such as gait speed and distance walked, as well as functional balance, compared to non-BWSTT (16, 17, 25–28). A study on 27 chronic stroke patients who underwent a 12-week BWSTT program in a community- based, outpatient rehabilitation setting found that patients had significant improvements in their walking endurance—the mean 6 MWT at baseline was 134 m (range: 12.2–315.5 m) while the mean 6 MWT after the BWSTT program was 244 m (range: 21.3–457 m), representing an overall improvement of 132% (range: 3–850%) (29). In another study of 100 stroke patients in an inpatient rehabilitation hospital, patients who were randomized to receive locomotor training with up to 40% BWS combined with treadmill training scored significantly higher than their counterparts who were randomized to receive locomotor training with full weight bearing (i.e., no BWS) in all clinical outcome measures including gait speed, distance walked and functional balance, after 6 weeks of training (26).

While BWSTT does appear to benefit stroke patients predominantly in walking capacity, its superiority over conventional gait-training strategies remains to be conclusively established. In a single-blind RCT of 97 sub-acute stroke patients involving 1-h training sessions (5 days per week for 4 weeks), patients who were randomized to receive BWSTT with up to 40% BWS demonstrated meaningful but similar improvements in gait speed, distance walked and balance ability as control patients who were randomized to receive conventional overground gait training (30) there were no significant differences between the 2 groups at the end of training. Srivastava et al. studied 45 chronic stroke patients randomized to 3 groups (conventional physiotherapy, treadmill training with full weight bearing or BWSTT with up to 40% BWS) for 30- min training sessions daily, 5 days per week for 4 weeks (28). Significant within-group improvements in outcome measures (gait speed, distance walked, and FAC score) were noted in all 3 groups. There were no significant between-group differences at the end of the training period, and gains were found to be sustained at 3-months follow-up across outcome measures and groups, with the exception of distance walked in the conventional physiotherapy group (28). While the outcomes were better in the BWSTT group, they were not significantly greater than those in the other 2 groups, leading the authors to conclude that BSWTT is not superior to conventional gait-training strategies for improving gait parameters.

However, current treadmills which are used to treat stroke patients have inherent features which cannot address safety concerns such as falling off the treadmill due to backward displacement if the patient cannot keep up with the belt speed, and these are summarized here: (i) a lack of automated safe speed progression; (ii) backward displacement with fixed belt speeds; and, (iii) absence of feedback to stroke patients with regard to foot placements on the moving belt; all of these are essential to mitigate fall risk.

Previously, Chua et al. tested a novel treadmill prototype, the Variable Speed and Sensing Treadmill (VASST I), which incorporated automated speed-control to adapt to patients' gait speeds and patterns, among other key features (31). Results of a pilot study in chronic stroke patients to assess the feasibility, safety and user acceptability of VASST I were promising, with improvements seen in distance walked, gait speed and balance immediately post-training and at follow-up (4 and 8 weeks, respectively) (31). The need and importance of novel ways of treadmill training in clinical practice to improve safety and reduce task complexity by ameliorating fall risks through backward displacements during treadmill training and reduction of frequent manual adjustments of speed progression by the physiotherapist are proposed in this study. Traditionally, pre-set non-variable treadmill speeds would need constant manual adjustment by physiotherapists standing at the front of the treadmill control panel, which increases the work of the therapist whose attention may be divided between manual assistance of the patient and treadmill speed adjustment and may not be able to focus on the patient's gait pattern or encouraging the patient. Furthermore, continuity of treadmill training could be disrupted due to repetitive stopping and starting of the treadmill should backward displacement of the patient occur. Through the addition of a safety harness and variable automated speed adjustment via sensors within the treadmill belt to trigger slowing should patients lag in catching up with a preset speed, improves the safety of treadmill training by reducing backward falls. Visual feedback through the user interface at the front of the treadmill gives real-time feedback to the patient with regards to individual foot position along the treadmill belt, allowing him to catch up with the preset speed and for speed to be automatically progressed if performance is consistent.

To investigate the combined efficacy of STT and BWSTT on gait performance, the VASST I (which was tested in subjects who needed only supervision and no manual aiding) was enhanced by incorporating an automated unweighting subsystem of up to 30 kg partial body weight support (BWS), with automated speed-control and sensing, resulting in the development of the VASST II (Variable Automated Speed and Sensing Treadmill II). The intent of VASST II was to meet the increased BWS requirements and enable treadmill training in stroke patients who needed minimal to moderate aid, who would usually not be considered to be able to train safely on conventional treadmills.

The aims of this exploratory study were to (i) study the feasibility and safety of the VASST II using a physiotherapist-supervised training protocol in an outpatient rehabilitation clinic and (ii) determine the VASST II's preliminary efficacy in sub-acute and chronic stroke patients with a predominant hemiplegic pattern of weakness. The primary outcome measure for this study was a change in distance walked (as measured by the 6 MWT) at the end of the training period.

## Materials and Methods

### Study Design

A prospective, single arm, open-label pilot feasibility study of subjects with sub-acute and chronic stroke was conducted in a tertiary rehabilitation center with close affiliations to an acute stroke unit. The technical development and hazard analyses of VASST II spanned 3 years (2015–2018), with a phase I open label clinical trial and follow-up being conducted thereafter over a period of 12 months (July 2018–June 2019). Ethical approval from institutional review boards was obtained prior to subject recruitment (NCT #01996137, https://www.clinicaltrials.gov; NHG DSRB 2013/1042). Written, informed consent was obtained from all subjects prior to all research interventions.

### Subjects

Subjects were identified and recruited consecutively, face-to-face, via attendances at the specialist outpatient clinic (SOC) of the tertiary rehabilitation center by the study team. The majority of these subjects previously underwent acute stroke rehabilitation in the inpatient rehabilitation unit of the rehabilitation center.

Subjects were included if they fulfilled the following inclusion criteria: (1) first-ever clinical stroke (ischaemic or haemorrhagic) confirmed on CT or MR brain imaging; (2) aged between 21 and 80 years; (3) stroke duration of >3 months post-stroke in the outpatient phase; (4) Functional Ambulation Category (FAC) score ≥2 (19); and (5) ability to walk overground at a self-selected speed of ≥0.2 m/s with or without walking aids, and/or lower limb orthoses for ≥50 m with moderate aid or less assistance.

Subjects were excluded if they had any of the following exclusion criteria: (1) cardiovascular conditions such as uncontrolled hypertension/hypotension, angina pectoris, recent myocardial infarction, congestive cardiac failure, known echocardiographic ejection fraction <40% within 3 months of stroke, chronic arrhythmias (e.g., atrial fibrillation) within 3 months of study screening, pacemaker, uncontrolled Diabetes Mellitus; (2) End stage illness (advanced malignancy), pregnancy or end stage renal failure with life expectancy of <6 months; (3) aphasia (i.e., inability to obey 2 step commands), communication disorders precluding understanding of instructions, cognitive impairment, dementia, untreated depression, or psychiatric disorder; (4) active lower limb arthritis, pain score (as measured on the Visual Analog Scale) >5/10, fixed joint deformities of the lower limb which would compromise safe ambulation on treadmill; (5) moderate to severe lower limb spasticity or spasms (Modified Ashworth Scale score >2); or (6) active trunk skin conditions, known abdominal aortic aneurysm, abdominal masses, or anticoagulation with Warfarin or Factor X inhibitors which could increase bleeding risk while using a gait harness.

### Technical Aspects of VASST II

**Figure 1:**
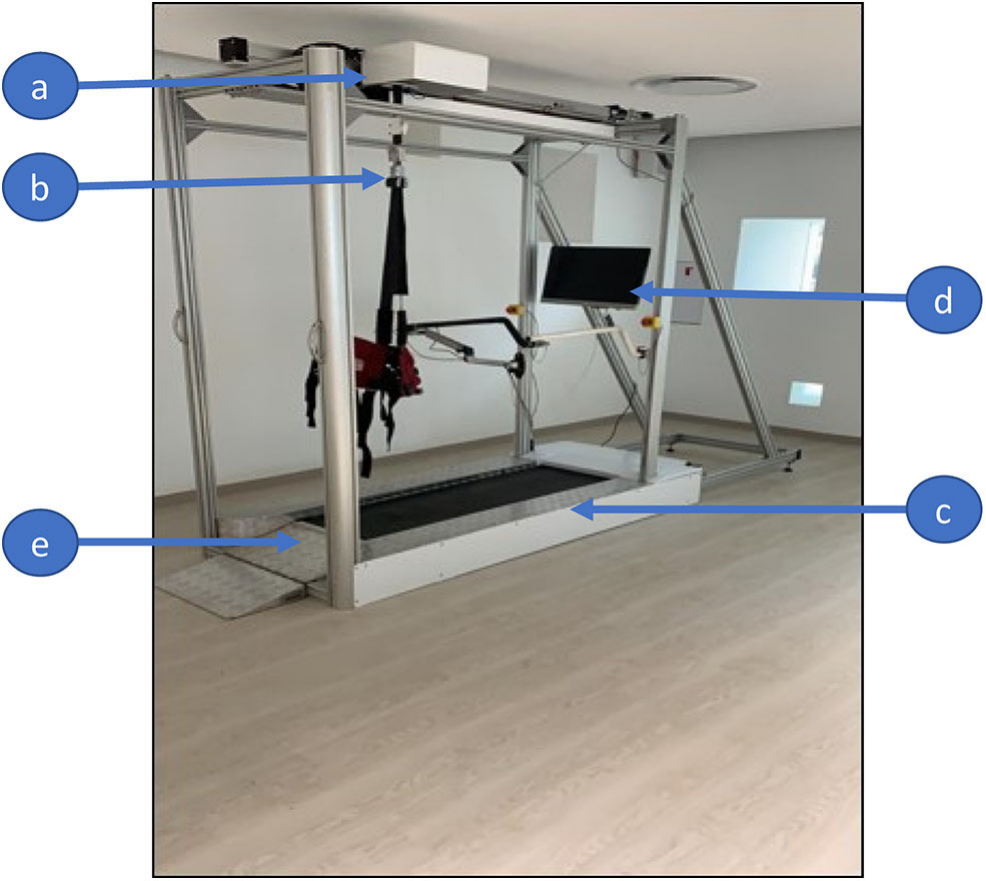
The Variable Automated Speed and Sensing Treadmill II (VASST II) is a semi-automated, integrated rehabilitation system with multiple sensors and microcontrollers. **(a)** Automatic control to reposition the harness horizontal position based on the subject's position on the walking belt; **(b)** automatic control to adjust the length of the supporting harness allowing subject unweighting up to 30 kg; **(c)** pre-programmed exercise parameters with a new set of sensors. The sensors' positions can be adjusted to match the subject's stride length; this optimizes and individualizes the training of the subject; **(d)** new software tools display real-time ambulatory gait speed, distance and time walked; **(e)** smaller width of the treadmill in VASST II (Length 3.65 m × Width 0.92 m × Height 2.58 m) compared to VASST I (Length 2.8 m × Width 1.6 m × Height 2.38 m).

The Variable Automated Speed and Sensing Treadmill II (VASST II) is a semi-automated, integrated rehabilitation system with multiple sensors and microcontrollers (Figure 1), based on enhancements from the VASST I, for which a detailed description is referenced here (32). Key features of VASST II are illustrated in Figure 1.

**Figure 2:**
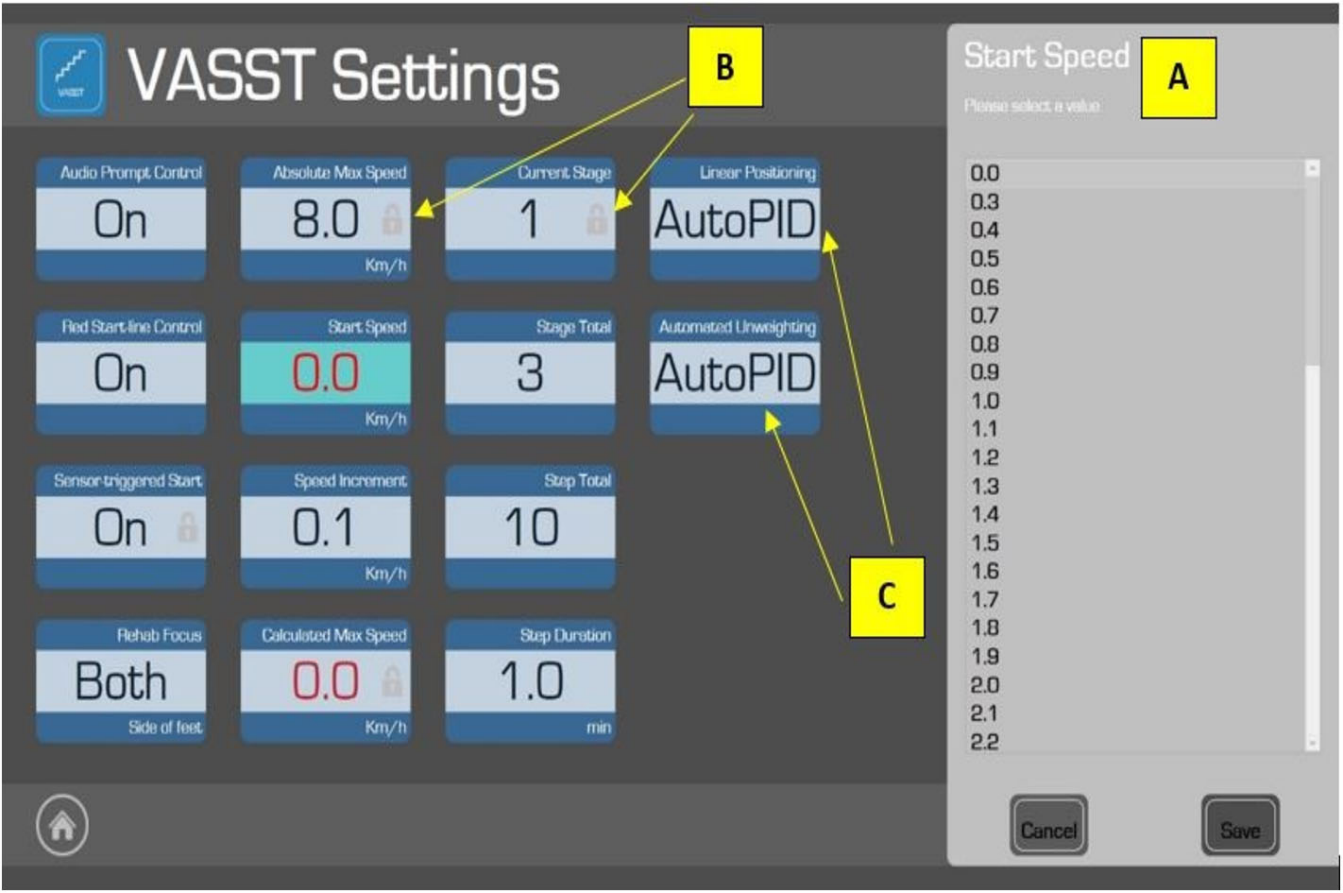
The “Settings” page in the VASST II which allows the supervising physiotherapist to adjust the exercise algorithm based on each patient's specific parameters. The Start Speed **(A)** of the treadmill is set by the physiotherapist. Certain features (e.g., Absolute Max Speed) carry a small padlock symbol **(B)** to indicate pre-programmed parameters which are fixed for subject safety. Adjustable parameters could be adjusted in this order: (1) *Audio Prompt Control*: to turn on/off the audio beep function; (2) *Red Start Line Control*: to turn on/off the red starting line indicator on the moving treadmill belt; (3) *Rehab Focus*: allows the physiotherapist to select which leg (right, left, or both) should be the focus of that particular exercise session (however, this function was disabled for VASST II); (4) *Start Speed*: set by the physiotherapist before each training epoch (10 min) based on the physiotherapist's clinical judgement and comfort level of the subject; (5) *Speed Increment*: while the default is 0.1 km/h, the supervising physiotherapist may adjust this to challenge the subject; (6) *Stage Total*: to set the number of training epochs (maximum of 5); (7) *Step Total*: to set the number of sections in each epoch; and (8) *Step Duration*: to adjust the duration of each section in each of the training epochs (the default duration is 1 min). Other parameters were non-adjustable, such as *Automated Unweighting* and *Linear Positioning*, where the values were set as AutoPID (Automatic Proportional–Integral–Derivative) **(C)**—this could enable or disable the automatic control function of the overhead harness suspension.

**Figure 3:**
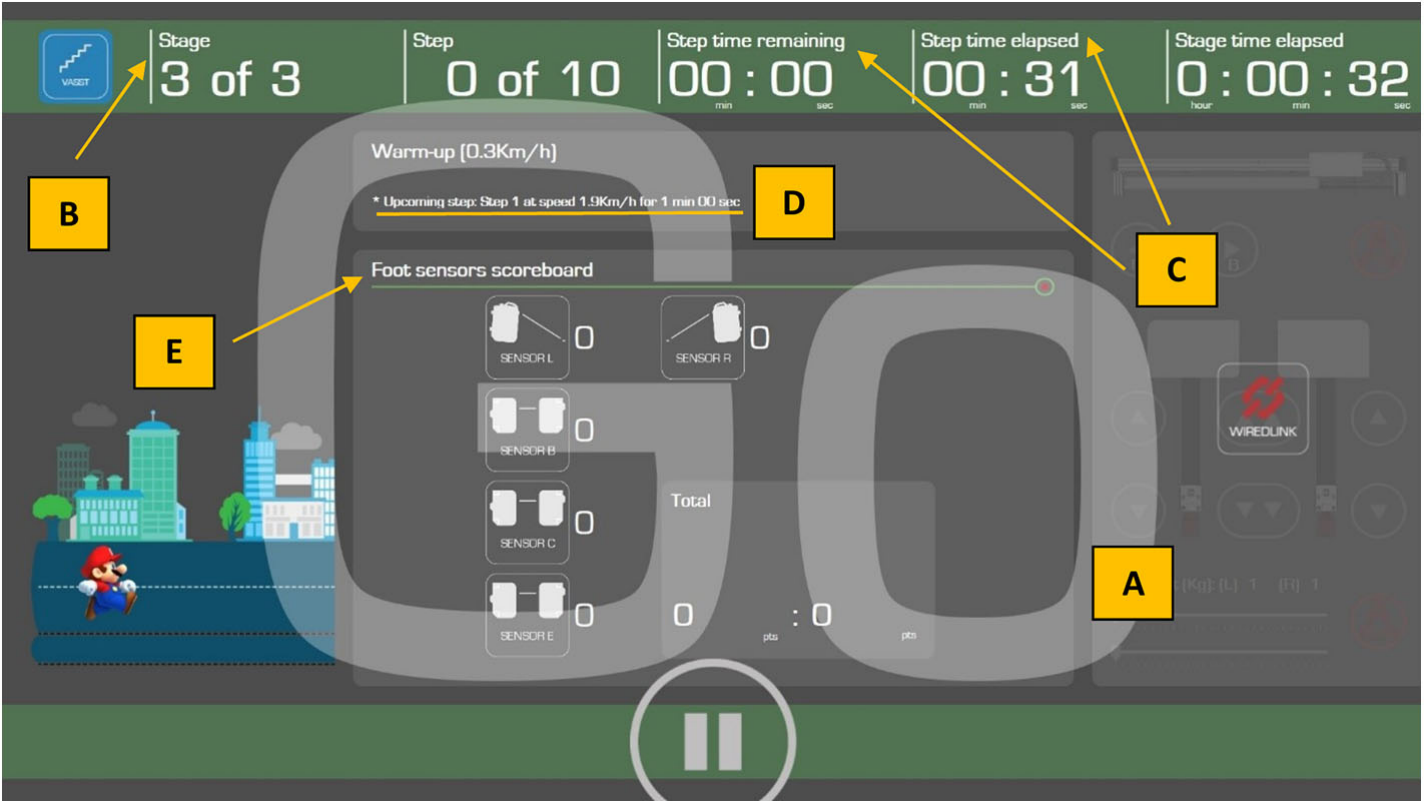
Screenshot of the visual feedback provided to study subjects on the 24-inch screen. Subjects are alerted to the impending start of the treadmill belt by visual cues [i.e., descending number order 3, 2, 1, and Go **(A)**, as well as audible beeps]. The training stage **(B)**, and elapsed or remaining training duration (minutes) **(C)**, are indicated in the top green banner. The upcoming speed of the treadmill belt **(D)** is also indicated in the first horizontal gray bar. Visual representations of the foot sensors in the middle gray section (*Foot sensors scoreboard*) **(E)** indicate positions of the left foot (*Sensor L*) and right foot (*Sensor R*) of the walking subjects and their respective positions on the treadmill belt. Foot position on these 2 front sensors (i.e., *Sensors R* and *L*) and *Sensor B* (second row) will increase the “Good” score to indicate to the subject that his/her foot placements are correct and he/she is performing the exercise well, while foot position on Sensor C (third row) will increase the “Bad” score to indicate to the subject that his/her foot placements are inaccurate, thus decreasing the efficiency of the exercise. Foot position on *Sensor E* (last row), apart from increasing the “Bad” score, will trigger an emergency stop of the treadmill. The final score is computed automatically by VASST II to determine the outcome (i.e., increase, decrease, or maintain speed for the next minute).

**Figure 4:**
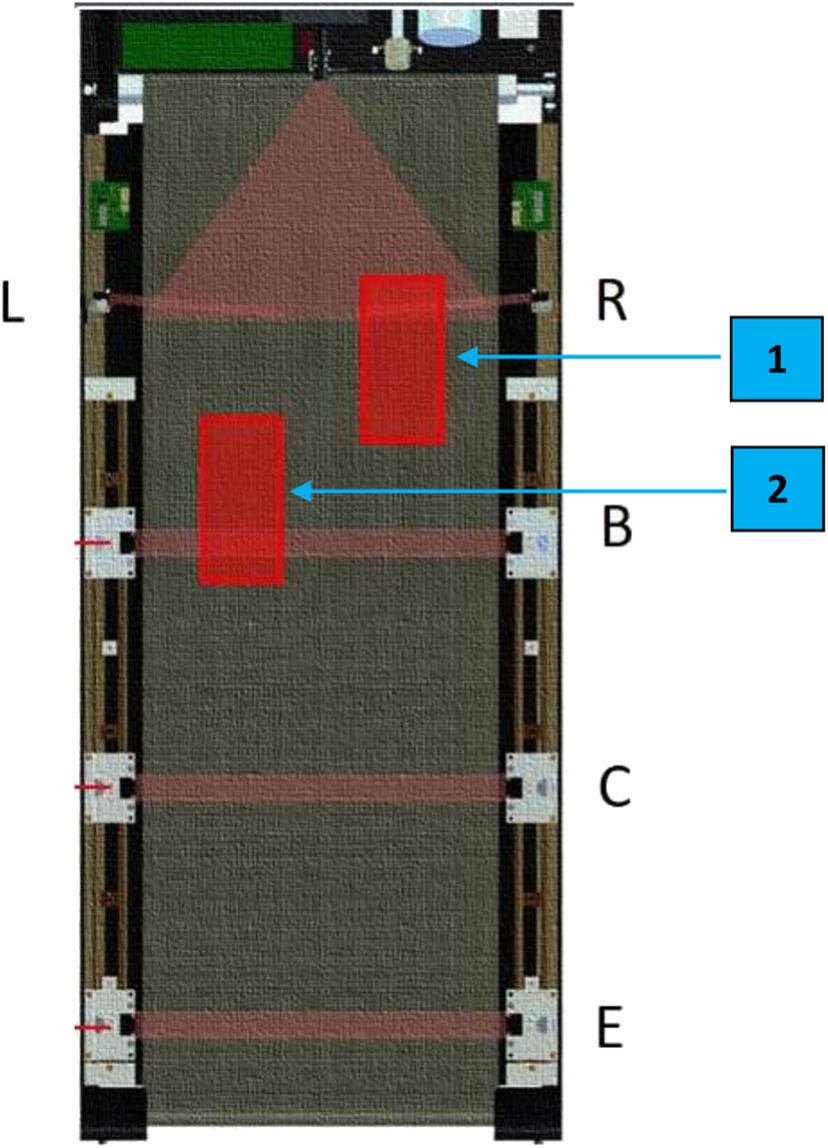
Illustration of the locations of the foot sensors on the VASST II treadmill belt. Proximal foot sensors, R (for the right foot) and L (for the left foot), are located at the front of the treadmill belt. Detection of foot positions on either of these 2 front sensors (e.g., if the right foot is at location 1) 60–70% of the time over 1 min would result in the treadmill belt speed increasing by 0.1 km/h for the next 1 min. If subjects are not able to keep up with the treadmill speed and begin to regress (e.g., if the left foot is at location 2), their foot positions would be detected on the distal foot sensors (B,C) which would result in the treadmill belt speed decreasing by 0.1 km/h for the next 1 min. If subjects continue to regress backwards at the lowered speed and their foot placement triggers the rear-most sensor (E), the treadmill would come to a halt; this safety feature of VASST II reduces the falls risk of subjects.

A standard rehabilitation treadmill, Mobility Research's GaitKeeper GK2000T® (33), was modified and enhanced in the following manner:

- The manufacturer's controller was replaced with a 32-bit (ARM4) microcontroller which interfaced directly with the treadmill's motor driver to control belt movement, store exercise parameters, and performance measurements during exercise and execute the exercise program using training algorithms tailored to each patient's parameters (Figure 2). These were pre-set by the treating physiotherapist. Data was uploaded to a personal computer at the end of the exercise. A 24-inch television screen provided enhanced visual feedback to the subject (Figure 3).
- Foot movements were detected by four cross-beam laser sensors placed across the treadmill. As described previously (in the VASST I prototype), laser beams could be interrupted by patient behavior (32). However, in the current VASST II device, the first set of cross- beam sensors were replaced by a set of sensors (Figure 4) which generated readings related to the distance walked. The sensors also differentiated between the subjects' legs, triggering the first set of sensor via its single reflective sensor, thus allowing a more detailed analysis of the cadence of the subject by comparing the number of steps made by the right foot and left foot.
- Automatic control of both the horizontal position of the overhead supporting system and vertical length of the support harness was facilitated by another set of 32-bit (ARM4) micro-controller. Such control of the horizontal position allowed the angle of the supporting harness's belt to be measured using a hall effect sensor whereby a positive output was generated by forward movement while a negative output was generated by backward movement; this allowed the automatic detection and control of the subject's location by the system. Force exerted by two load cells located on the overhead suspension controlled the vertical length of the harness—Pulling down on the harness resulted in its extension while remaining still resulted in the automatic retraction of the harness by the system to a set point.

### Study Flow

Subject training sessions using the VASST II, as well as post-training follow-up sessions, were conducted at the rehabilitation outpatient clinic.

A standard VASST II training protocol was employed for all 11 subjects, which consisted of a total of 15 h-long sessions of treadmill training on the VASST II over 5 consecutive weeks (i.e., 3 h-long sessions weekly). All VASST II treadmill training sessions were closely monitored by a senior physiotherapist and a bioengineer.

Each hour-long session comprised the following, in sequence:

- 5 min of warm up exercise and limb stretches;
- 5 min to suit up in the light harness;
- Commencement of walking without arm supports for 5 min on VASST II at a self-selected steady speed;
- In total, 40 min of three 10-min epochs each of variable gait speed training incorporating rest breaks (3–5 min) according to perceived rating of exertion, target heart rate, and patient fatigue; and
- 5 min of cool down exercise.

A speed-dependent treadmill training protocol was employed in the following manner:

1. Subjects were allowed to hold either one of the treadmill handlebars at the start of each training session until they were confident of their balance, before releasing their hand-holds.
2. Subjects were instructed to keep walking toward the front of the treadmill system in order to trigger the 2 proximal foot sensor positions, R and L (Figure 4).
3. If these foot placements were detected 60–70% of the time over 1 min, speed of the VASST II automatically increased by 0.1 km/h for the next 1 min.
4. If the subject could not keep up with the pre-set VASST II training speed and the distal undesired 2 foot sensors, B and C (Figure 4), were triggered 60–70% of the time during the 1 min, the treadmill speed was maintained at the same speed for the next minute.
5. If the subject was still unable to keep up during this minute, the speed was automatically adjusted downwards by 0.1 km/h for the next 1 min.
6. If subjects continued to regress backwards and the rear-most foot sensor, E (Figure 4), was triggered, the treadmill was halted to avoid a potential fall.
7. Such progressions were repeated until the training epoch was completed.
8. Subjects were allowed to rest for 3–5 min prior to commencement of the next training epoch.
9. The degree of body weight support (BWS) needed for each subject was clinically determined by treating physiotherapists at the commencement of each training session and reassessed 2 more times prior to each new epoch of training (*refer to iv above*). The level of BWS up to 30 kg was adjusted based on each subjects' baseline performance and adaptation to VASST II training. Subjects were weighed at Week 0 prior to BWS prescription. For subjects who walked with minimal and moderate aid, the baseline BWS level was set at ~10–25% of their body weight, respectively, based on the assessment of the physiotherapist prior to training. Supervised walkers who did not require manual aiding were started on 5% BWS initially. During VASST II training, if subjects were walking smoothly and able to keep up with the set treadmill training speed during that epoch, the level of BWS was reduced by 1 kg after each completed epoch. If subjects' performance indicated difficulties (such as excessive holding onto the handle bars, inability to keep to the front half of the treadmill belt, loss of balance, or frequent stops during training), the degree of BWS was maintained or increased by 1 kg. The mean BWS per session was recorded for each subject and consisted of the average of 2–3 readings per session, depending on the number of BWS adjustments needed by each subject. BWS as a percentage of the subject's baseline bodyweight was also computed.
10. Subjects did not need to be manually assisted by therapists who were stationed beside the VASST II device. Apart from automated speed reduction or progression, therapists were able to intervene by steadying hands on the body harness or manually operating emergency stop buttons if needed. This was followed by 5 min of cool down, which marked the end of the VASST II training session.

Subjects did not receive any other physiotherapy-based interventions during the 5-week VASST II treadmill training period; this was to prevent any possible injuries related to intensive locomotor training as well as to evaluate the immediate post-training effects of the VASST II trial. The types of rehabilitation therapies prior to VASST II training or during the follow-up phase for 19 weeks were not documented as part of the study protocol.

### Safety Monitoring

Prior to commencing VASST II training, the target heart rate (THR) limit for each subject was calculated using the following formula: THR = {[HR peak (220) – HR (at rest)] × [60–70%] + HR (at rest)}. Each subject's blood pressure and heart rate were measured prior to and at the end of VASST II treadmill training. Training sessions were temporarily halted if subjects felt unwell or experienced any of the following: chest pain, excessive exertional dyspnoea, and severe limb pain.

### Outcomes Assessment

Independent physiotherapists who were not involved in VASST II treadmill training sessions performed all outcome assessments. Subjects were assessed at the following fixed time intervals: Week 0 (baseline), Week 3 (mid-VASST II training period), Week 6 (post-VASST II training period), Week 12 (1st follow- up), and Week 24 (2nd follow-up). A total of 3 physiotherapists were involved in the study interventions.

#### Primary Outcome Measures

The primary outcome measures were (i) walking capacity (distance walked), assessed by the 6 minute walk test (6 MWT) which was performed according to 2002 ATS guidelines (34), and (ii) gait speed on self-selected overground walking, as assessed by the 10-meter walk test (10 MWT). The 6 MWT distance walked was measured using a single, timed distance on self-selected level ground indoors (loop of 21.5 m) (35). The 10 MWT measured walking speed over the middle 10 m of a 14-m course (21). Scores for the 10 MWT were calculated based on the average of two trials. All tests were done without physically aiding the subjects, but with consistent use of walking aids or orthoses as required by subjects.

#### Secondary Outcome Measures

Secondary outcome measures included (i) ambulation ability, as assessed by the Functional Ambulation Category (FAC) score; (ii) overall balance ability, as assessed by the Berg Balance Scale (BBS) score; (iii) occurrence of adverse events such as pain and falls; and (iv) subjective feedback from study subjects and physiotherapists.

- *Ambulation ability*: The FAC is a 6-point scale (score range: 0–5, whereby a score of 0 indicates a non-functional ambulator, and a score of 5 indicates an independent ambulator) which assesses ambulation status by observing how much human support a patient requires to ambulate a distance of 10 feet (3.05 m), regardless of whether a personal assistive device is used or not (19).
- *Overall balance ability*: The BBS, generally considered to be the gold standard in functional balance assessment of older adults, is a widely used 14-item scale (score range: 0–56, whereby a score of 0–20 indicates a wheelchair-bound patient, 21–40 indicates a patient able to walk with assistance, and 41–56 indicates an independent walker) (36).
- *Adverse events*: Prior to the start of each treadmill training session, the presence of prolonged muscle aches or joint pains lasting more than a day was recorded. The Visual Analog Score (VAS) for pain (pain VAS), a 10-point scale (score range: 0–100, whereby a score of 0 indicates no pain while a score of 100 indicates worst imaginable pain) which allows patients to describe pain intensity, was used to measure the presence of pain at the end of each treadmill training session. Falls occurring during treadmill training sessions and during follow up visits were also recorded.
- *Subjective feedback*: User acceptability of VASST II for both study subjects and the 3 physiotherapists who were involved in VASST II training were assessed using short, self-rated questionnaires administered at the end of the 5-week training period (Week 6).

Study subjects were asked the following questions:

1. In your opinion, did you benefit from training on VASST II in the past 4 weeks?
2. To what extent did training on VASST II help your current walking ability?
3. The greatest benefit after VASST II training?
4. Would you like more sessions on VASST II?
5. Please rate your training experience.

The supervising physiotherapist was asked the following questions:

1. In your opinion, did VASST II improve safety aspects of treadmill training?
2. Did VASST II help to reduce manpower requirements for safe treadmill training?
3. Would you like to use VASST II in your treatment of future patients?

### Statistical Analyses

Data were analyzed using IBM SPSS version 19.0 (IBM Corp, Armonk, NY). Descriptive statistics were used to summarize the baseline characteristics of subjects. Normality of the data was assessed using skewness, kurtosis and histogram. The Friedman test was used to explore the effectiveness of VASST II training over different time points (Weeks 0, 3, 6, 12, and 24), and Wilcoxon Signed Rank test with *p*-value adjustments were further carried to identify the significant change at specific time points. All the tests were two sided.

## Results

### Baseline Data (Week 0)

**Figure 5:**
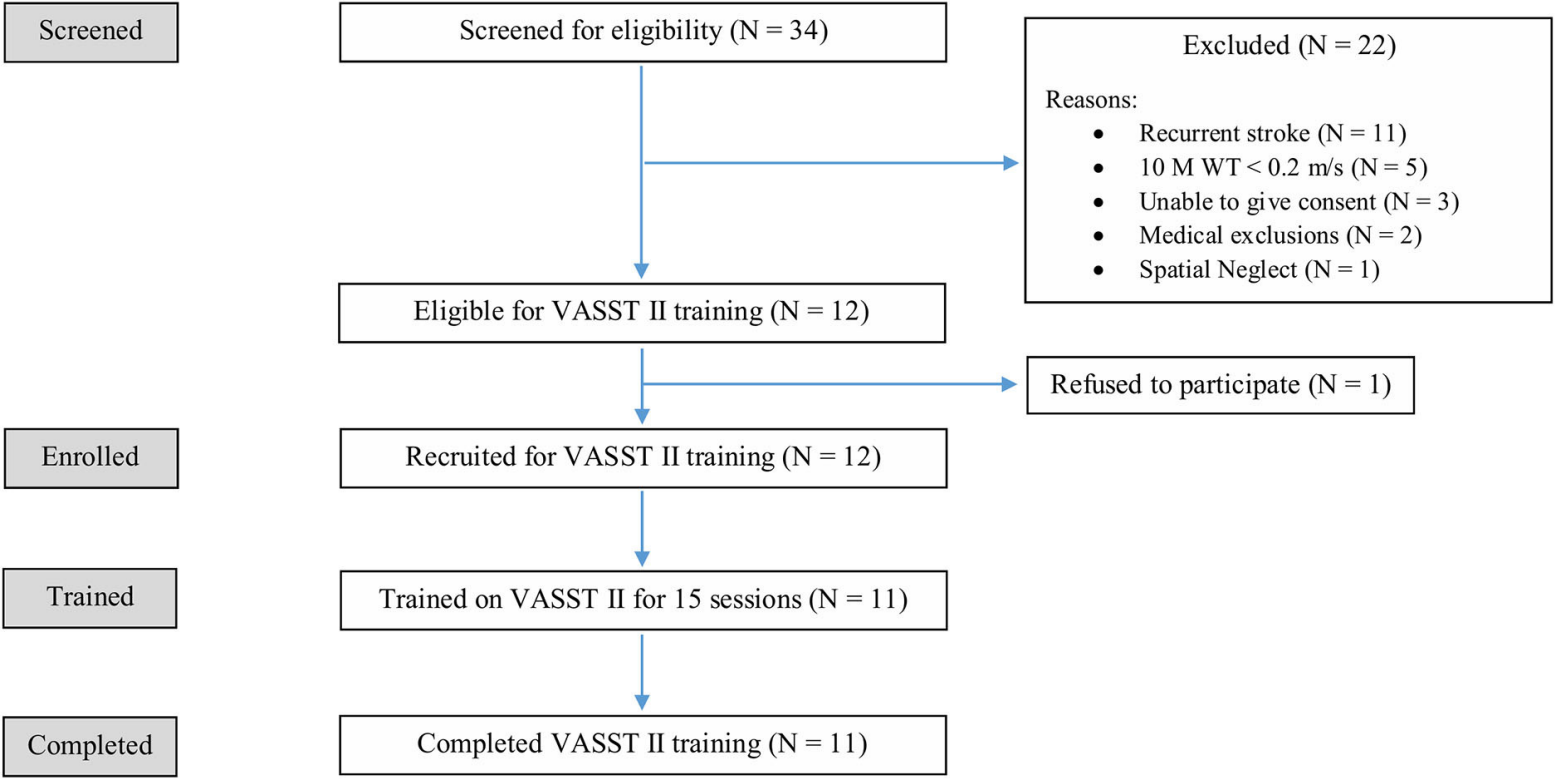
Flow diagram of the VASST II recruitment and study process.

Of the initial 34 patients screened, 22 patients did not meet the study eligibility criteria and 1 patient declined consent. In all, a total of 11 eligible patients were enrolled as study subjects. Figure 5 shows the flow diagram of the recruitment and study process. All 11 enrolled subjects completed 15 VASST training sessions each, and all follow-up visits. There were no drop outs.

The baseline demographic and clinical characteristics of the study subjects are shown in Table 1. The median (IQR) age of the study subjects was 53.0 (22) years. Seven of the 11 subjects (63.6%) were male and 8 of the 11 (72.7%) suffered haemorrhagic stroke. The median (IQR) duration post-stroke was 524 (811) days; 45.5% (5 of the 11 subjects) were within the subacute phase of stroke (≤180 days) while the remainder (54.5%) were in the chronic phase (>180 days). More than 81% of patients used walking aids (such as quadruped sticks and single canes), while slightly over half used orthoses (e.g., ankle foot orthoses, ankle braces, etc.). Despite the need for support devices post-stroke, 63.6% (7/11) were able to ambulate independently on level ground (FAC score = 5). The mean ± SD BBS score, out of a total score of 56, was 40 ± 10. The baseline mean ± SD gait speed, determined by the 10 MWT, was 0.37 ± 0.18 m/s while the baseline mean ± SD distance walked, determined by the 6 MWT, was 114 ± 50.9 m. With regard to previous treadmill walking experience, only 18.2%(2 of the 11 subjects) had previous exposure to conventional treadmill training as they needed supervision for overground walking (FAC score = 4). The majority (81.8%) had no previous treadmill training as they were unable to use conventional treadmills due to the high level of assistance (minimal to moderate) they required for safe usage of treadmills in view of their gait symmetry.

**Table 1:** Baseline demographic and clinical characteristics of study subjects (*N* = 11).

**Baseline characteristic**	**Value**
Age, years^a^	53.0 (22)
Duration post-stroke at study initiation, days^a^	524 (811)
Distance walked, m^b^^,^^c^	114 ± 50.9
Gait speed, m/s^b^^,^^d^	0.37 ± 0.18
BBS score, /56^b^	40 ± 10
Gender, *n* (%)	
- Male	7 (63.6%)
- Female	4 (36.4%)
Type of stroke, *n* (%)	
- Infarct	3 (27.3)
- Intracerebral hemorrhage	8 (72.7)
Side of stroke, *n* (%)	
- Left	9 (81.8)
- Right	2 (18.2)
Affected side, *n* (%)	
- Left	2 (18.2)
- Right	9 (81.8)
Stroke management, *n* (%)	
- Conservative	5 (45.5)
- Neurosurgery	6 (54.5)
Use of walking aids, *n* (%)	
- No	2 (18.2)
- Yes	9 (81.8)
Use of orthoses, *n* (%)	
- No	5 (45.5)
- Yes	6 (54.5)
FAC score, *n* (%)	
- 2	1 (9.1)
- 3	3 (27.3)
- 4	0 (0)
- 5	7 (63.6)

*BBS, Berg Balance Scale; FAC, Functional Ambulation Category*.

a *Values are presented as Median (IQR)*.

b *Values are presented as Mean ± SD*.

c *Determined using the 6- minute walk test (6 MWT)*.

d *Determined using the 10-meter walk test (10 MWT)*.

The overall changes in the primary outcome measures (6 MWT and 10 MWT) and secondary outcome measures (BBS score and FAC score) at the different study time points (Weeks 0, 3, 6, 12, and 24) are summarized in Table 2. Subjects' baseline bodyweight and progression of BWS by session over time are presented in Supplementary Tables S1, S2. After initial adaptation to VASST II training, most subjects, with the exception of one, demonstrated a steady decline in the amount of BWS needed as treadmill training progressed over the 15 sessions.

**Table 2:** Outcome measures by time point (*N* = 11).

**Outcome measure**	**Time point**	**Mean ± SD**	**Median (IQR)**	***P*-value**
Distance walked (6 MWT), m	Week 0 (baseline)	114 ± 50.89	108 (104)	<0.001^*^
	Week 3 (mid-VASST II training)	124.55 ± 65.55	127 (120)	
	Week 6 (post-VASST II training)	158.91 ± 88.69	152 (162)	
	Week 12 (1st follow-up)	170.18 ± 104.63	136 (214)	
	Week 24 (2nd follow-up)	172.82 ± 121.01	114 (178)	
Gait speed (10 MWT), m/s	Week 0 (baseline)	0.37 ± 0.18	0.32 (0.30)	0.001^*^
	Week 3 (Mid-VASST II training)	0.40 ± 0.22	0.37 (0.43)	
	Week 6 (post-VASST II training)	0.49 ± 0.30	0.40 (0.61)	
	Week 12 (1st follow-up)	0.54 ± 0.35	0.38 (0.69)	
	Week 24 (2nd follow-up)	0.51 ± 0.38	0.30 (0.52)	
BBS score, /56	Week 0 (baseline)	40 ±10	43 (16)	<0.001^*^
	Week 3 (mid-VASST II training)	42 ± 10	44 (16)	
	Week 6 (post-VASST II training)	42 ± 10	46 (15)	
	Week 12 (1st follow-up)	45 ± 9	47 (15)	
	Week 24 (2nd follow-up)	42 ± 10	43 (13)	
FAC score	Week 0 (baseline)	4 ± 1	5 (2)	0.121
	Week 3 (mid-VASST II training)	4 ± 1	5 (2)	
	Week 6 (post-VASST II training)	4 ± 1	5 (1)	
	Week 12 (1st follow-up)	5 ± 1	5 (0)	
	Week 24 (2nd follow-up)	4 ± 1	5 (2)	

*Friedman test was performed*.

* *Significant p < 0.05*.

*6 MWT, 6 minute walk test; 10 MWT, 10 meter walk test; BBS, Berg Balance Scale; FAC, Functional Ambulation Category*.

### Post-VASST II End of Training Results (Week 6)

**Figure 6:**
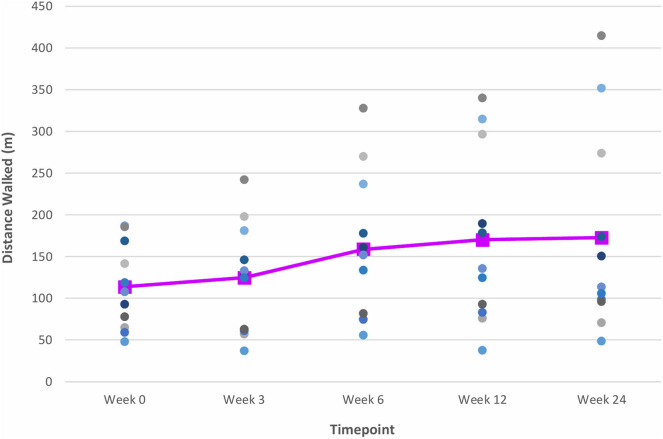
Time series plot showing the primary outcome measure of distance walked (as measured by the 6 MWT). Each circle • represents a study subject. A trend line (–) has been superimposed to illustrate the change in the mean ± SD distance walked from baseline pre-intervention (i.e., Week 0) to final follow-up post-intervention (i.e., Week 24).

**Figure 7:**
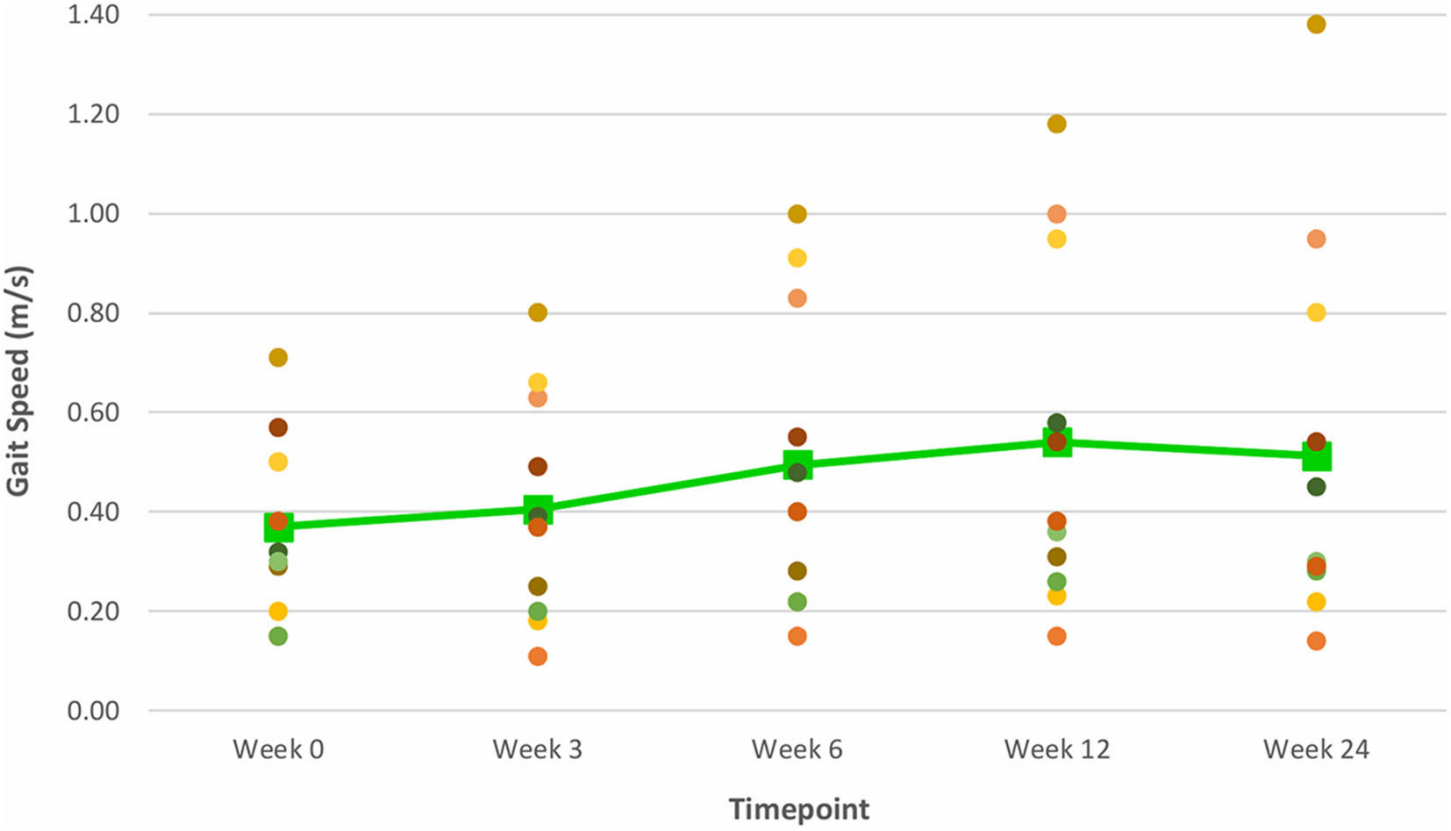
Time series plot showing the primary outcome measure of gait speed (as measured by the 10 MWT). Each circle • represents a study subject. A trend line (–) has been superimposed to illustrate the change in the mean ± SD gait speed from baseline pre-intervention (i.e., Week 0) to final follow-up post-intervention (i.e., Week 24).

**Figure 8:**
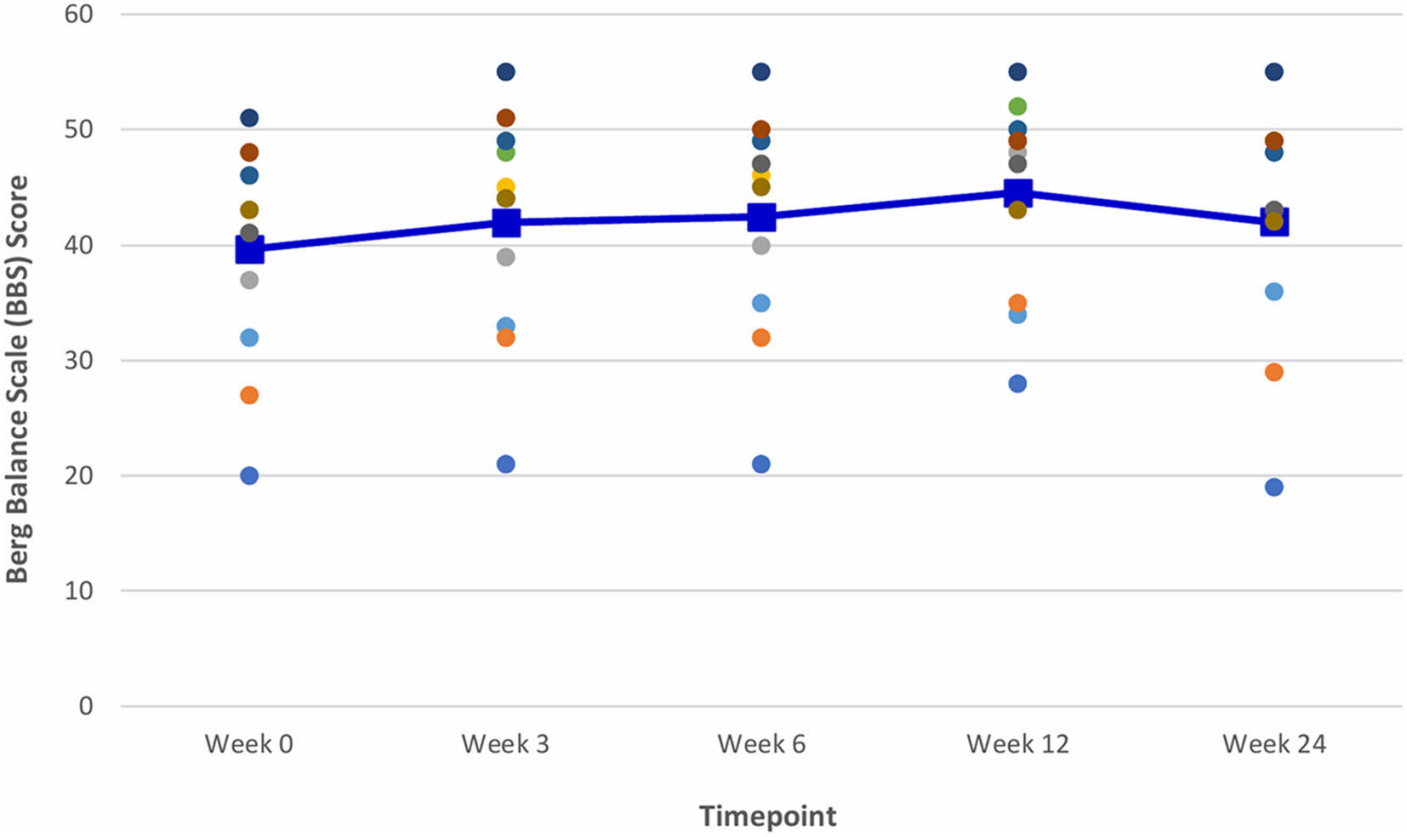
Time series plot showing the secondary outcome measure of Balance Berg Scale (BBS) score. Each circle • represents a study subject. A trend line (–) has been superimposed to illustrate the change in the mean ± SD BBS score from baseline pre-intervention (i.e., Week 0) to final follow- up post-intervention (i.e., Week 24).

**Figure 9:**
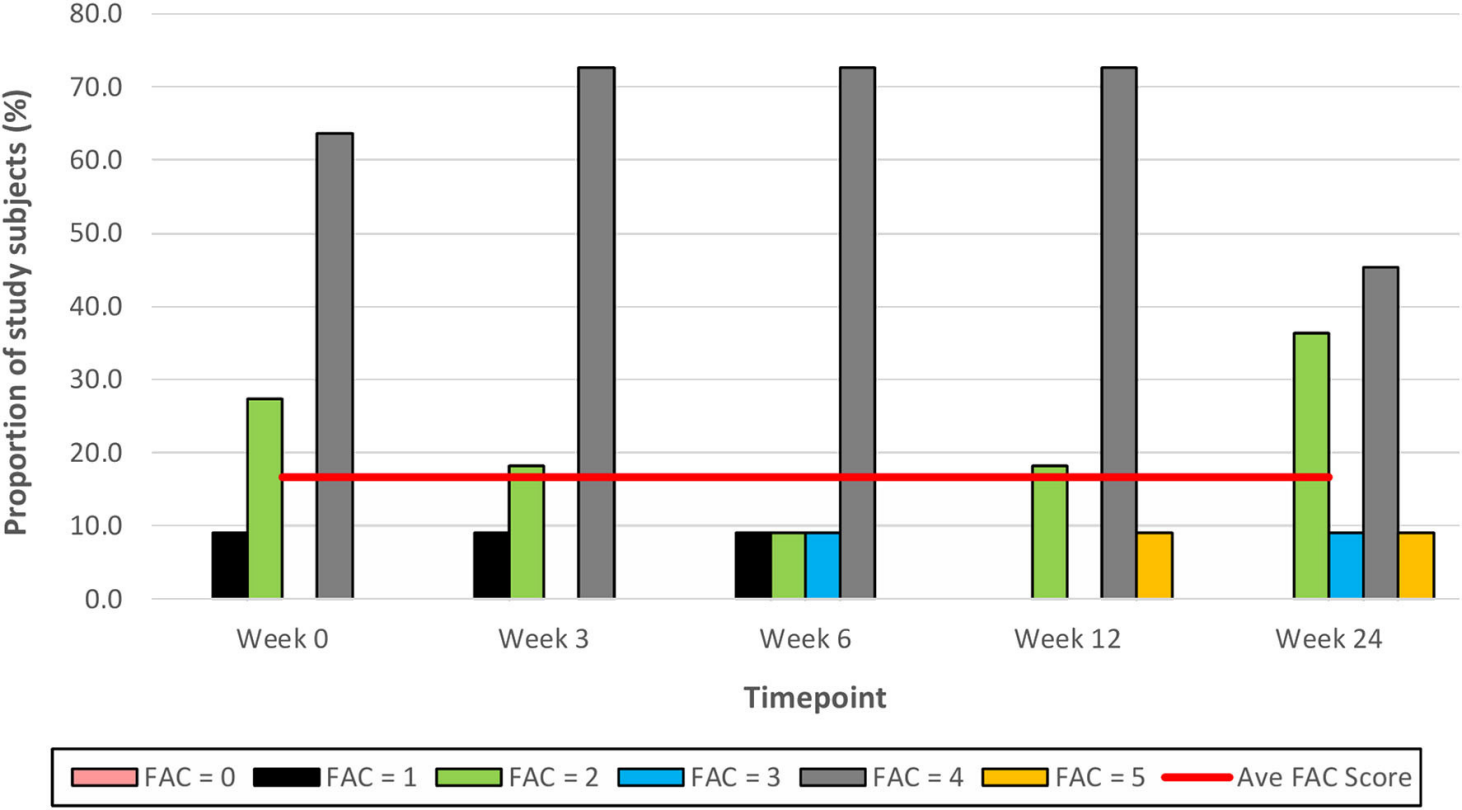
Time series plot showing the secondary outcome measure of Functional Ambulation Category (FAC) score. Each bar represents a FAC score (range 0–5, with 0 representing a non-functional ambulation and 5 representing independent ambulation). A trend line (–) has been superimposed to illustrate the change in the mean ± SD FAC score from baseline pre-intervention (i.e., Week 0) to final follow-up post-intervention (i.e., Week 24).

At Week 6, after 5 consecutive weeks of treadmill training, there were improvements observed in both primary outcome measures of distance walked (measured by the 6 MWT) (Figure 6) and gait speed (measured by the 10 MWT) (Figure 7), when compared to Week 0 (baseline). The mean ± SD improvement in the 6 MWT was 44.9 ± 49.2 m; *P* = 0.003, representing a 39.4% increase in distance walked (Table 3). The mean ± SD improvement in the 10 MWT was 0.12 ± 0.15 m/s; *P* = 0.016, representing a 35.1% increase in gait speed (Table 3). A significant gain of 5% was also observed in the BBS score (mean ± SD of 2.82 ± 1.89 points; *P* = 0.003; Figure 8). The improvement in FAC score was neither significant at Week 6 nor at subsequent time points (Figure 9).

**Table 3:** Within-group differences by time point (*N* = 11).

**Outcome measure**	**Change in mean ± *SD***	**Change in %**	***P*-value**
**Distance walked (6 MWT), m**			
Week 3–Week 0	10.55 ± 28.00	9.25	0.449
Week 6–Week 0	44.91 ± 49.16	39.4	0.003^*^
Week 12–Week 0	56.18 ± 63.84	49.3	0.007^*^
Week 12–Week 6	11.27 ± 26.92	7.09	0.213
Week 24–Week 0	58.82 ± 80.37	51.6	0.013^*^
Week 24–Week 6	13.91 ± 46.76	8.75	0.789
Week 24–Week 12	2.64 ± 32.01	1.55	0.790
**Gait speed (10 MWT), m/s**			
Week 3–Week 0	0.03 ± 0.08	9.34	0.182
Week 6–Week 0	0.12 ± 0.15	33.7	0.016
Week 12–Week 0	0.17 ± 0.21	45.9	0.018
Week 12–Week 6	0.05 ± 0.07	9.19	0.082
Week 24–Week 0	0.14 ± 0.24	37.8	0.086
Week 24–Week 6	0.02 ± 0.14	3.13	0.878
Week 24–Week 12	−0.03 ± 0.09	−5.56	0.153
**BBS score, /56**			
Week 3–Week 0	2 ± 1	5.73	0.005^*^
Week 6–Week 0	3 ± 2	7.11	0.003^*^
Week 12–Week 0	5 ± 3	11.9	0.005^*^
Week 12–Week 6	2 ± 3	4.49	0.084
Week 24–Week 0	2 ± 2	5.96	0.012
Week 24–Week 6	0 ± 2	−1.07	0.234
Week 24–Week 12	−3 ± 3	−5.32	0.030
**FAC score**			
Week 3–Week 0	0 ± 1	4.35	0.317
Week 6–Week 0	0 ± 1	6.52	0.180
Week 12–Week 0	1 ± 1	19.57	0.063
Week 12–Week 6	1 ± 0	12.2	0.083
Week 24–Week 0	0 ± 1	−4.35	0.705
Week 24–Week 6	0 ± 1	−10.2	0.458
Week 24–Week 12	0 ± 1	−20.0	0.102

*6 MWT, 6 minute walk test; 10 MWT, 10 meter walk test; BBS, Berg Balance Scale; FAC, Functional Ambulation Category*.

*Wilcoxon Signed Rank test was performed*.

* *Significant p < 0.007 (0.05/7 = 0.007)*.

### Post-VASST II Follow-Up Results (Week 12)

The gains observed in the 6 MWT, 10 MWT and BBS score from baseline to Week 6 continued to improve at Week 12 (Figures 6–8). The mean ± SD improvement at Week 12 in the 6 MWT was 56.2 ± 63.8 m; *P* = 0.007, a 49.3% increase from baseline (Week 0) (Table 3). Similarly, there was a 45.9% increase from baseline to Week 12 in the 10 MWT (mean ± SD of 0.17 ± 0.21 m/s; *P* = 0.018; Table 3). In addition, there was a 11.9% improvement in the BBS score from baseline to Week 12 (mean ± SD of 5 ± 3 points; *P* = 0.005; Table 3).

### Post-VASST II Follow-Up Results (Week 24)

The improvements observed in the 6 MWT and BBS score at Week 6 and 12 were sustained at the end of the study period (Figures 6, 8). Compared to baseline (Week 0), there was a 51.6% increase in the 6 MWT (mean ± SD of 58.8 ± 80.4 m; *P* = 0.013; Table 3). There was also an increase of 5.96% in the BBS score (mean ± SD of 2 ± 2 points; *P* = 0.012) from baseline to Week 24 (Table 3). Although there was a 37.8% increase in the 10 MWT from baseline to Week 24 (Figure 7) this improvement was not statistically significant (mean ± SD of 0.14 ± 0.24; *P* = 0.086; Table 3).

### Adverse Events

There were no reports of VASST II-training related serious adverse events or falls (Week 0 to Week 5). Three (27.3%) of the 11 study subjects experienced transient exercise-related muscle and joint aches which resolved spontaneously within 24 h of the VASST II training session. During the follow-up period from Week 6 to Week 24, there was 1 report of each of the following: stroke (Week 12), minor fall during BBS assessment (Week 12), epileptic seizure (Week 24), and knee injury (Week 24). However, none of these was attributable to the VASST II training. No subjects dropped out during the follow-up period.

### User Acceptability of VASST II

Overall, study subjects rated their training experience on VASST II positively. All 11 (100%) study subjects found VASST II training to be beneficial in improving their walking ability over 5 weeks of training, and were keen to undergo further VASST II training sessions (Supplementary Table S3). Nine (81.8%) of the 11 study subjects rated their VASST II training experience as good or excellent. Nine (81.8%) and 2 (18.2%) of subjects indicated that VASST II training greatly improved or somewhat improved their current walking ability, respectively (Supplementary Table S3).

The three supervising physiotherapists rated VASST II favorably, reporting that it improved the safety aspects of treadmill training in all patients (100%), reduced manpower requirements for 7 patients (63.6%), and desired its future use (100%) all patients' training sessions (Supplementary Table S4).

## Discussion

Overall, this was a positive exploratory feasibility study for VASST II, a hybrid treadmill device incorporating automated sensing, speed dependent and partial BWS up to 30 kg, in the rehabilitation of post-stroke patients in an ambulatory setting. All 11 study subjects completed 15 1-h sessions of treadmill training over 5 weeks, with no serious adverse events or drop outs reported during the 5-week training period or 19-week follow-up period. Notably, more than 80% of subjects who had no prior treadmill experience prior to VASST II training, as their need for minimal to moderate aid during overground ambulation precluded safe and feasible training in conventional manual treadmills, successfully completed all 15 sessions of VASST II training. Hence the original intent for such dependent walkers to be feasibly trained in an integrated automated variable speed and adjustable BWS treadmill was achieved in this exploratory clinical study. Both subacute and chronic patients, who were fairly equally represented in this sample completed training on VASST II.

At the end of the training period, there were significant and meaningful improvements in walking capacity and gait speed, and significant gains in balance ability, compared to baseline. These improvements were sustained and continued to improve up to 19 weeks post-cessation of training. Usability of VASST II was also highly rated by both study subjects and supervising physiotherapists for its perceived benefits in improving walking capacity and stability, lower limb strength and balance, reducing manpower demands, and safety during training. Application of the safety harness and treadmill device set-up time were also qualitatively acceptable to both study subjects and supervising physiotherapists.

Post-training cessation, mean improvements from baseline in walking capacity, as measured by the 6 MWT, of 44.9, 56.2, and 58.8 m were noted at Weeks 6, 12, and 24, respectively. These improvements far exceeded the MCID range of 34.4–44 m for meaningful improvement in distance walked, suggesting that the 5-week VASST II treadmill training resulted in clinically important and meaningful benefits for walking ability post-stroke up to 24 weeks (22, 23).

Mean improvements from baseline gait speeds of 0.12, 0.17, and 0.14 m/s, as measured by the 10 MWT, were also noted at Weeks 6, 12 and 24 post-training respectively. While the improvement at Week 6 exceeded the threshold for a small meaningful change in gait speed (0.06 m/s), only the improvements at Weeks 12 and 24 met the MCID range of 0.14–0.16 m/s for substantial meaningful change in gait speed (20, 21). Such gains attest to the ability of VASST II treadmill training to produce clinically significant improvements in gait speed in chronic post-stroke patients which persisted for 19 weeks after training had ceased. These motor gains are widely thought to be due to repetitive stepping, motor patterning and increased gait cycle practice induced by sensorimotor stimulation from treadmill training drives cerebral reorganization through use-dependent neuroplasticity (37, 38). It is also postulated that the benefits in walking distance and speed could be a result of new features of VASST II such as (i) pre-programmed exercise parameters with sensors which allowed the device to mount a speed challenge to study subjects depending on their walking ability; (ii) automated speed progression; and (iii) provision of visual feedback to subjects on their progress relating to gait speed, distance walked and time taken.

Mean improvements from baseline of 2, 3, and 5 points in the BBS score were noted at Weeks 3, 6, and 12, respectively. Although statistically significant, the clinical significance of these improvements following VASST II training is uncertain as there is a paucity of literature data on the MCID threshold for the BBS score in chronic stroke patients.

With regard to gains in distance walked, gait speed, and respective MCID thresholds attained, results from our sample were comparable with that reported by early studies on treadmill training (12, 19). However, the baseline characteristics of this current study sample imply a more severely impaired population as we recruited those with a minimum gait speed of ≥0.2 m/s and FAC score ≥2; subjects' baseline 6 MWT was also lower, at 114 m. With regard to treadmill training parameters, the majority of studies involving BWSTT report varying amounts of BWS (25–40%), as well as a range of treatment intensities and durations (4–12 weeks, with most individual sessions lasting 30–40 min). Our BWS (10–25% at commencement) and treadmill training protocol (40 min of training on VASST II within a 60-min session) were comparable, however, we employed a relatively shorter training duration with moderate intensity rather than high intensity (i.e., 3 times per week instead of 5 times per week) as we wanted to factor in recovery time between training sessions to prevent training-related fatigue and limb pain. Self-limiting joint and muscle pain were reported by a quarter of the study sample (3 of 11 subjects) at Week 6.

The current study sample mostly demonstrated a trend to lowered BWS as training progressed, with 81.8% (9 of 11 subjects) requiring <10% BWS at the end of 15 training sessions (Supplementary Tables S1, S2). We postulate that this could be related to the integration of BWS and variable automated speed. The sustained and improved gains up to 19 weeks post-training suggests that this protocol was able to achieve short-term sustained gains with meaningful clinical impact, and possibly implies positive neuroplastic change. At study conclusion, gait parameters of the study sample indicated that significant locomotor disability remained (compared to pre-stroke levels), as the majority were still limited community ambulators (6 MWT: 172 m and 10 MWT: 0.51 m/s). It is possible that a longer treatment duration at higher training intensity could portend greater gains and further potential for community ambulation (26, 29, 30).

In the current study, there were no improvements in study subjects' walking independence (as measured by the FAC score) post-treadmill training; the mean FAC score remained unchanged at 4 throughout the study duration. This is consistent with the results of a systematic review which found that treadmill training, regardless of BWS, did not increase stroke patients' chances of walking independently, compared with patients who did not receive such training [Risk Difference −0.00, 95% confidence interval −0.02 to 0.02; *P* = 0.94] (14). While the FAC measure has excellent reliability, good concurrent and predictive validity, and good responsiveness in patients with stroke, it has been associated with reduced responsiveness among individuals with a lower level of functional ability as well as large ceiling effects (39–41). This could be a plausible explanation for the lack of improvement in the FAC score of patients in this current study.

It was noteworthy from the qualitative assessment that VASST II was rated highly both by study subjects, who found the training to be beneficial and useful, as well as by the 3 supervising physiotherapists, who found that it reduced manpower requirements and risk of falls (Supplementary Tables S3, S4). In a rapidly aging population with high stroke burden, training with VASST II has the potential to meet intensive rehabilitation training needs without a concomitant increase in trained labor force or training related serious side effects.

### Comparative Analyses of VASST II and VASST I

The positive outcomes of this study lend further support to the findings of a previous study based on an earlier iteration, the VASST I. VASST II permits a wider range of stroke walking abilities to be treated by safe supervised treadmill training safely (31). In spite of similar inclusion and exclusion criteria between VASST I and II devices, key differences between VASST I and VASST II study subjects were shown in their baseline walking abilities; VASST I study subjects had to be able to ambulate at least 150 m with contact guard or supervision (FAC score >4) on level ground at a self- selected walking speed of ≥0.1 m/s with or without walking aids or orthoses in order to be eligible for study participation. For the current study however, subjects who were more functionally impaired at baseline (FAC score ≥2), with similar self-selected walking speed and distance could be included due to the additional feature of partial BWS (up to 30 kg) in the VASST II. When the study populations between VASST I and II were compared, these differences were exemplified in the baseline gait speed (VASST I: 0.69 m/s vs. VASST II: 0.37 m/s), baseline distance walked (VASST I: 178.3 m vs. VASST II: 114 m), and baseline BBS score (VASST I: 48 points vs. VASST II: 40 points) (Supplementary Table S5).

After 12 sessions of treadmill training over 4 weeks, VASST I study subjects experienced significant 428 improvements in the 6 MWT (54.3 ± 30.9 m; *P* = 0.005), 10 MWT (0.06 ± 0.08 m/s; *P* = 0.037), and BBS score (2 ± 2; *P* = 0.005) (31). These motor gains were sustained up to 4 weeks post-cessation of training (Δ in 6 MWT: 55.9 ± 31.8 m, *P* = 0.005; Δ in 10 MWT: 0.12 ± 0.1 m/s, *P* = 0.013; and Δ in BBS score: 2 ± 2, *P* = 0.01). Of note, VASST II study subjects underwent 1 additional week (increase of 25%) of treadmill training compared to VASST I study subjects. This longer training period could have contributed to the increased quantum and sustainability of the motor gains observed in the current study, despite VASST II having less competent walkers compared to VASST I. Moreover, the additional feature of partial BWS (up to 30 kg) in the enhanced version of the treadmill device was also a contributing factor.

### Limitations

This study had the following limiting factors: (i) In terms of baseline demographics of the study sample, the mean age of the subjects was 47 years, and nearly three-quarter (72.7%) were diagnosed with intracerebral hemorrhage. This was not representative of the local stroke population, which has a mean age of 68 years, and one-fifth (20%) of patients have the haemorrhagic stroke sub-type (42). This dissimilarity thus significantly limits the generalizability of the study findings to the local stroke population; (ii) There was no comparator or control group to assess VASST II's efficacy against usual treadmill training or overground gait training strategies; (iii) the study could have been underpowered to detect further differences due to the small sample size of 11 patients. However, as the objectives of this exploratory clinical trial were to evaluate the feasibility, safety, and user acceptance of a novel, hybrid device, an open-label design with a relatively small sample size and shorter training period (compared to other studies) was intentional in order to allow further technical iterations; (iv) Variations in ambulation capabilities of subjects, training protocols, and follow-up durations across studies limit direct head-on-head comparisons with VASST II study findings; and (v) There was a lack of objective gait parameters (such as spatial and kinematic data).

### Future Studies

A randomized, controlled trial with a larger sample size of adequate statistical power and longer training period to compare VASST II against standard treadmill or overground training techniques would be necessary to establish the superiority and usability of VASST II over conventional strategies in improving locomotor abilities after stroke.

## Conclusion

Ambulant, chronic stroke patients who received training on VASST II, a hybrid automated speed-sensing treadmill system with partial BWS mechanism, experienced significant improvements in walking capacity, gait speed, and overall balance ability at the end of training. The majority of subjects trained had not been able to previously train on manual treadmills due to significant hemiplegic weakness. At primary end point, both walking capacity and gait speed greatly exceeded the respective MCID thresholds, demonstrating potential benefits to subjects' performance in daily life. Improvements in walking capacity and gait speed were sustained 19 weeks following the cessation of VASST II. User acceptance of VASST II was high, with both study patients and supervising physiotherapists rating it favorably in terms of user experience and safety. Insights from this pilot study will facilitate further enhancements and technical iterations to allow it to be used in the future by rehabilitation clinics to train hemiplegic gait.

## Data Availability Statement

All datasets presented in this study are included in the article/Supplementary Material.

## Ethics Statement

The studies involving human participants were reviewed and approved by National Healthcare Group domain specific ethics board. The patients/participants provided their written informed consent to participate in this study.

## Author Contributions

KaC and CW contributed to the conceptual development and hazard analysis of the VASST II research prototype device, and were involved in the following: obtained institutional ethics approval, design and conduct of the clinical trial, subject recruitment, informed consent-taking, monitoring of subjects during the intervention and follow-up periods, clinical data collection, statistical analysis and reporting of results, and formulation of the discussion and conclusion. WLim and PL contributed to the conceptual development and hazard analysis of the VASST II research prototype, design and conduct of the clinical trial, training and supervision of all research subjects during the intervention period, monitoring of subjects during the follow-up period, and clinical data collection. CL performed the statistical analyses and generated the tabular and graphical data. WO, WLiu, KuC, CH, and JC contributed to the conceptual development and hazard analysis of the VASST II research prototype during the laboratory phase, and were involved in monitoring during the clinical trial phase and technical data collection. All authors read, reviewed, and approved the final manuscript.

## Conflict of Interest

The authors declare that the research was conducted in the absence of any commercial or financial relationships that could be construed as a potential conflict of interest.
