# Supplementary material for: An Exploratory Clinical Study on an Automated, Speed-Sensing Treadmill Prototype With Partial Body Weight Support for Hemiparetic Gait Rehabilitation in Subacute and Chronic Stroke Patients
Source: Front Neurol. 2020 Jul 24;11:747. doi: 10.3389/fneur.2020.00747 (PMC7394021; doi:10.3389/fneur.2020.00747)
Supplement: Supplementary file 1 [file Data_Sheet_1.docx]

Supplementary Material

# Supplementary Tables


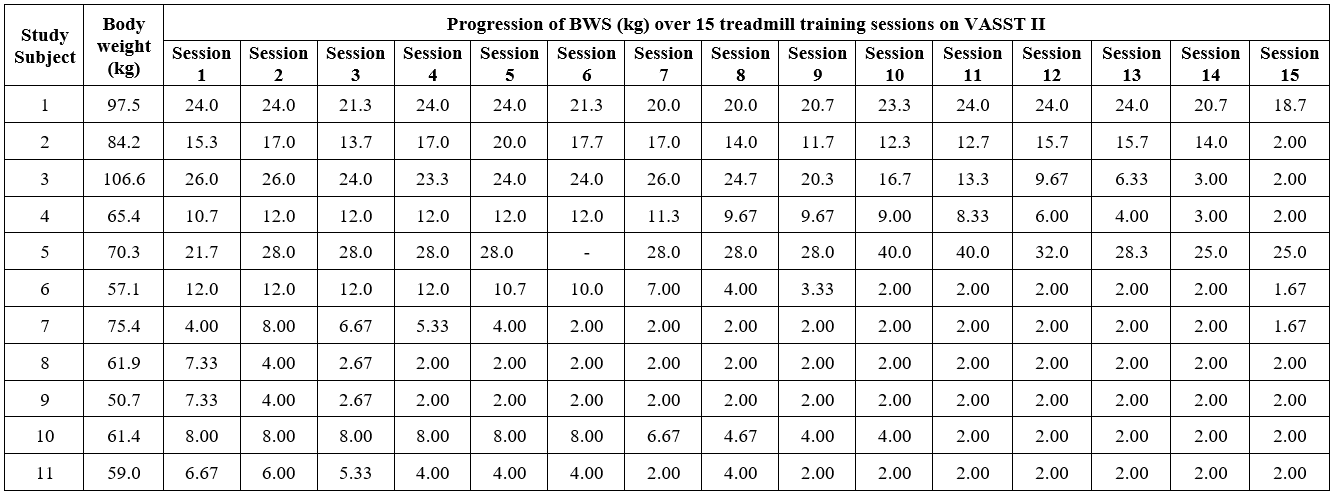
**Supplementary Table S1.** Subjects’ (N = 11) baseline body weight (kg) and the progression of bodyweight support (BWS/kg) over the course of the VASST II treadmill training period (total of 15 sessions over 5 weeks).


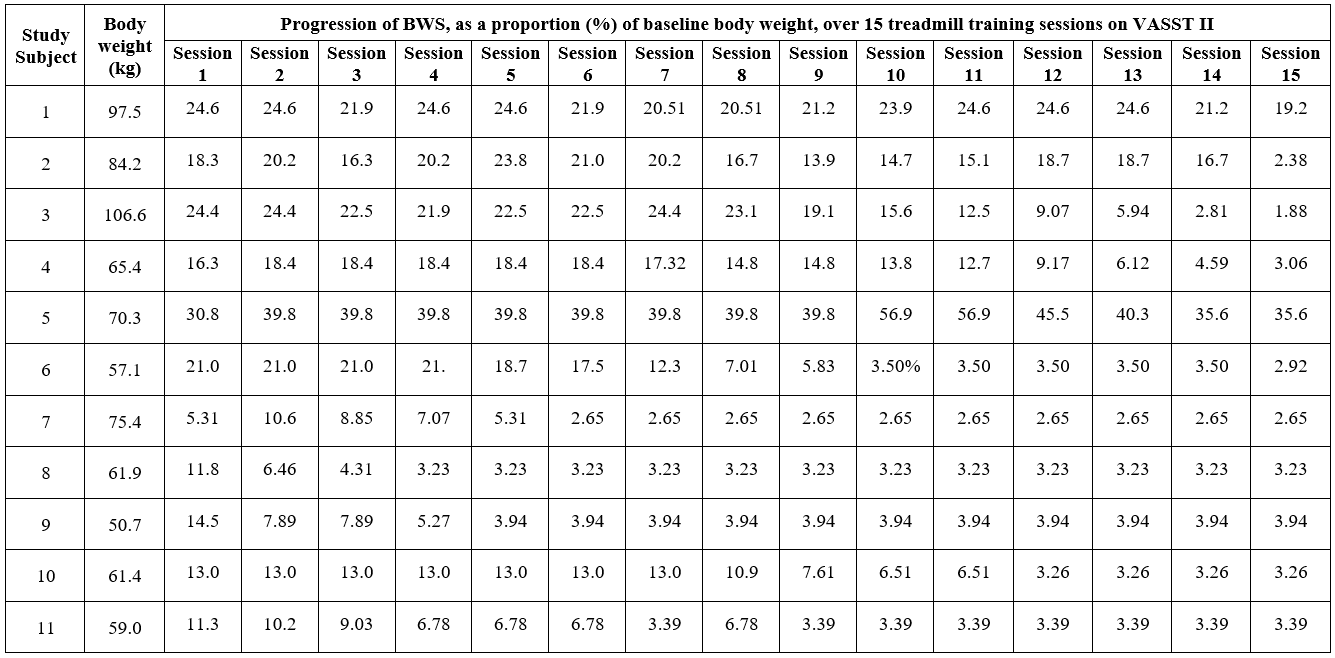
**Supplementary Table S2.** Progression of bodyweight support (BWS /kg), as a proportion of subjects’ (N = 11) baseline body weight (kg), over the course of the VASST II treadmill training period (total of 15 sessions over 5 weeks).

**Supplementary Table S3.** Self-rated questionnaire administered to patients (N = 11) at the end of the training period and the frequency of their responses.

| **Question** | **Response Options** | **Frequency (%)** |
| --- | --- | --- |
| 1. In your opinion, did you benefit from training on VASST II in the past 5 weeks? | No  Yes | 0 (0 %)  11 (100 %) |
| 1. To what extent did training on VASST II help your current walking ability? | Worsened  Not at all  Neutral  Somewhat improved  Greatly improved | 0 (0 %)  0 (0 %)  0 (0 %)  9 (81.8 %)  2 (18.2 %) |
| 1. The greatest benefit after VASST II training: | Walk faster  Walk more  Legs feel stronger  More balanced | 3 (27.3 %)  2 (18.2 %)  4 (36.4 %)  2 (18.2 %) |
| 1. Would you like more sessions on VASST II? | No  Yes | 0 (0 %)  11 (100 %) |
| 1. Please rate your training experience: | Poor  Satisfactory  Good  Excellent | 1 (9.1 %)  1 (9.1 %)  3 (27.3 %)  6 (54.5 %) |

**Supplementary Table S4.** Self-rated questionnaire administered to the supervising physiotherapists at the end of the training period to gather feedback on their experiences in using VASST II for each study subject (N = 11) and the frequency of their responses.

| **Question** | **Response Options** | **Frequency (%)** |
| --- | --- | --- |
| 1. In your opinion, did VASST II improve safety aspects of treadmill training? | No  Yes  Neutral | 0 (0 %)  11 (100 %)  0 (0 %) |
| 1. Did VASST II help to reduce manpower requirements for safe treadmill training? | No  Yes  Neutral | 3 (27.3 %)  7 (63.6 %)  1 (9.1 %) |
| 1. Would you like to use VASST II in your treatment of future patients? | No  Yes  Neutral | 0 (0 %)  11 (100 %)  0 (0 %) |

**Supplementary Table S5.** Comparison of baseline characteristics between VASST I and VASST II study subjects.

| **Baseline Characteristic** | **VASST I study subjects**  **(N = 10)** | **VASST II study subjects**  **(N = 11)** |
| --- | --- | --- |
| Distance walked, m ^a^ | 178.3 ± 84.0 | 114 ± 50.9 |
| Gait speed, m/s ^b^ | 0.69 ± 0.29 | 0.37 ± 0.18 |
| BBS score, /56 | 48 ± 3 | 40 ± 10 |
| FAC score | 5 ± 0 | 4 ± 1 |

*BBS, Berg Balance Scale; FAC, Functional Ambulation Category.*

*Values are presented as Mean ± SD.*

*^a^ Determined using the 6 minute walk test (6 MWT).*

*^b^ Determined using the 10 metre walk test (10 MWT).*
